# Di-μ-benzoato-κ^3^ *O*,*O*′:*O*;κ^3^ *O*:*O*,*O*′-bis­[(acetato-κ*O*)(1,10-phenanthroline-κ^2^ *N*,*N*′)lead(II)] dihydrate

**DOI:** 10.1107/S1600536809025896

**Published:** 2009-07-11

**Authors:** Junli Gao, Xiaopeng Xuan

**Affiliations:** a Department of Chemistry, Henan Normal University, Xinxiang 453007, People’s Republic of China

## Abstract

The title compound, [Pb_2_(CH_3_COO)_2_(C_7_H_5_O_2_)_2_(C_12_H_8_N_2_)_2_]·2H_2_O, consists of dimeric units built up around a crystallographic centre of symmetry and two non-coordinating water mol­ecules. Each Pb^II^ unit is six-coordinated by a bidentate 1,10-phenanthroline (phen) ligand, a monodentate acetate anion and a bidentate benzoate anion, which also acts as a bridge linking the two Pb^II^ atoms. The crystal packing is stabilized by O—H⋯O hydrogen bonds and by π–π inter­actions between the phen rings of neighboring mol­ecules, with a centroid–centroid distance of 3.577 (3) Å.

## Related literature

For information on the coordination chemistry of lead, see: Shimoni-Livny *et al.* (1998 ▶). For related structures, see: Li & Yang (2004 ▶); Xuan *et al.* (2008 ▶); Xuan & Zhao (2007 ▶); Zhao *et al.* (2007 ▶); Zhu *et al.* (2004 ▶). 
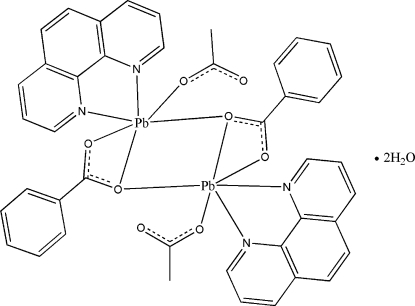


## Experimental

### 

#### Crystal data

[Pb_2_(C_2_H_3_O_2_)_2_(C_7_H_5_O_2_)_2_(C_12_H_8_N_2_)_2_]·2H_2_O
*M* *_r_* = 1171.15
Monoclinic, 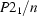

*a* = 11.809 (4) Å
*b* = 13.910 (5) Å
*c* = 12.290 (4) Å
β = 107.392 (4)°
*V* = 1926.5 (11) Å^3^
*Z* = 2
Mo *K*α radiation
μ = 8.79 mm^−1^
*T* = 294 K
0.11 × 0.07 × 0.05 mm

#### Data collection

Bruker SMART CCD area-detector diffractometer
Absorption correction: multi-scan (*SADABS*; Bruker, 1997 ▶) *T*_min_ = 0.445, *T*_max_ = 0.668
16820 measured reflections
4417 independent reflections
3343 reflections with *I* > 2σ(*I*)
*R*_int_ = 0.044

#### Refinement

*R*[*F* ^2^ > 2σ(*F* ^2^)] = 0.029
*wR*(*F* ^2^) = 0.057
*S* = 1.03
4417 reflections
263 parameters
18 restraints
H-atom parameters constrained
Δρ_max_ = 0.73 e Å^−3^
Δρ_min_ = −0.85 e Å^−3^

### 

Data collection: *SMART* (Bruker, 1997 ▶); cell refinement: *SAINT* (Bruker, 1997 ▶); data reduction: *SAINT*; program(s) used to solve structure: *SHELXS97* (Sheldrick, 2008 ▶); program(s) used to refine structure: *SHELXL97* (Sheldrick, 2008 ▶); molecular graphics: *SHELXTL* (Sheldrick, 2008 ▶); software used to prepare material for publication: *publCIF* (Westrip, 2009 ▶).

## Supplementary Material

Crystal structure: contains datablocks I, global. DOI: 10.1107/S1600536809025896/sj2634sup1.cif

Structure factors: contains datablocks I. DOI: 10.1107/S1600536809025896/sj2634Isup2.hkl

Additional supplementary materials:  crystallographic information; 3D view; checkCIF report

## Figures and Tables

**Table 1:** Selected bond lengths (Å)

Pb1—O3	2.399 (3)
Pb1—O2	2.426 (3)
Pb1—O1	2.565 (3)
Pb1—O1^i^	2.828 (3)
Pb1—N2	2.619 (4)
Pb1—N1	2.688 (4)

Symmetry code: (i) 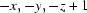.

**Table 2:** Hydrogen-bond geometry (Å, °)

*D*—H⋯*A*	*D*—H	H⋯*A*	*D*⋯*A*	*D*—H⋯*A*
O5—H2*W*⋯O3^ii^	0.83	2.15	2.958 (5)	166
O5—H1*W*⋯O4	0.83	2.11	2.928 (5)	169

Symmetry code: (ii) 
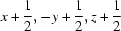
.

**Fig. 1.**
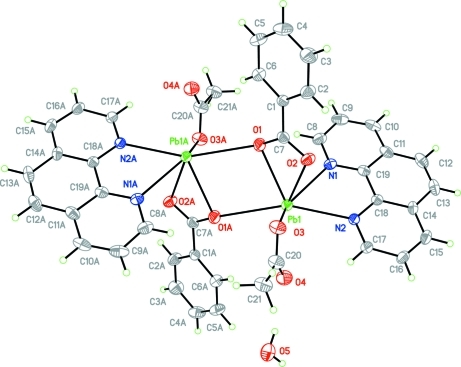
The molecular structure of the title compound, with atom labels and 30% probability displacement ellipsoids for non-H atoms. [Symmetry code for atoms labelled A: -x, -y, 1 - z].

**Fig. 2.**
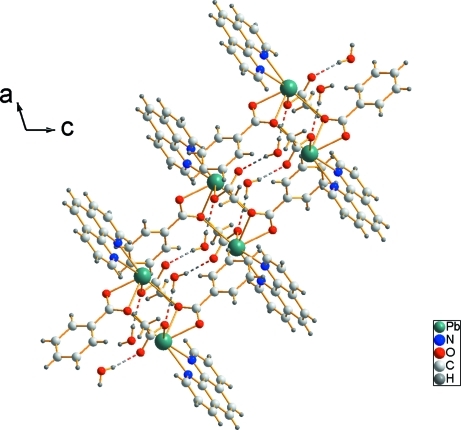
Part of the crystal structure of (I), showing the hydrogen-bonding (dashed lines) interactions.

**Fig. 3.**
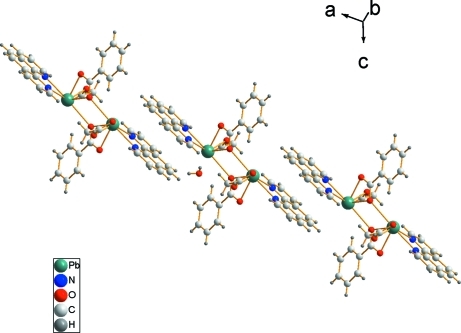
π-π interactions between the aromatic rings of the title compound.

## supplementary crystallographic information

### Comment

Because of the increasing impact of the toxic heavy metal lead on the natural environment, the coordination behavior of lead ion has received more and more attention. Lead(II) is capable of exhibiting a variable coordination number and geometry with or without a stereochemically active lone pair of electrons (Shimoni-Livny *et al.* 1998). Among such compounds, a number of centrosymmetric dinuclear lead(II) compounds with 1,10-phenanthroline (phen) or its derivatives and oxygen donor ligands have been structurally characterized (Li & Yang, 2004, Xuan *et al.* 2008, Xuan & Zhao, 2007, Zhao *et al.* 2007, Zhu *et al.* 2004,). Recently, we obtained the title lead(II) complex containing two different kinds of anions, by the reaction of lead acetate, sodium benzoate and phen in ethanol/water mixtures.

The crystal structure of the title compound consists of dimeric units [Pb_2_(C_2_H_3_O_2_)_2_(C_7_H_5_O_2_)_2_(C_12_H_8_N_2_)_2_], related by a crystallographic inversion centre (Fig. 1), and two uncoordinated water molecules. Both the acetate and benzoate anions are coordinated to each Pb(II) atom and a carboxylate oxygen of each benzoate anion forms a bridge between the two inversion related lead atoms. Each lead atom is chelated by the two N atoms of phen with Pb—N distances of 2.619 (4), and 2.688 (4) Å, three carbonyl oxygen atoms of two benzoate anions and one carbonyl oxygen atoms of an acetate anion. The weak Pb—O bridging interactions form a four-membered Pb_2_O_2_ quadrilateral with a Pb—Pb separation of 4.289 (5) Å.

The crystal structure is stabilized by intermolecular O—H···O hydrogen bonds (Table 1 and Figure 2). The uncoordinated water molecules participate in hydrogen bonding to oxygen atoms of the acetate anions. The crystal packing is further stabilized by π-π stacking interactions between adjacent phen molecules. The centroid-centroid distance between *Cg*1 (N1/C8—C11/C19) and *Cg*2 (N2/C14—C18)[symmetry code: 1 - *x*, -*y*, 1 - *z*] is 3.575 (3) Å.

### Experimental

A solution (10 ml) of ethanol containing 1,10-phenanthroline (0.5 mmol) and sodium benzoate (1 mmol) was added slowly to a aqueous solution (10 ml) containing lead acetate trihydrate (0.5 mmol). The mixture was refluxed for 5 h and the resulting white precipitate was filtered. Block-like single crystals were obtained by slow evaporation of the filtrate at room temperature after five days.

### Refinement

The carbon-bound H atoms were placed in calculated positions and were included in the refinement in the riding model approximation, with C—H = 0.93 Å and *U_iso_*(H) = 1.2*U_eq_*(C aromatic). The water H atoms were restrained at O—H = 0.83 Å with *U_iso_*(H) = 1.5*U_eq_*(O).

### Crystal data

**Table:**

[Pb_2_(C_2_H_3_O_2_)_2_(C_7_H_5_O_2_)_2_(C_12_H_8_N_2_)_2_]·2H_2_O	*F*(000) = 1120
*M**_r_* = 1171.15	*D*_x_ = 2.019 Mg m^−^^3^
Monoclinic, *P*2_1_/*n*	Mo *K*α radiation, λ = 0.71073 Å
Hall symbol: -P 2yn	Cell parameters from 3638 reflections
*a* = 11.809 (4) Å	θ = 2.3–23.1°
*b* = 13.910 (5) Å	µ = 8.79 mm^−^^1^
*c* = 12.290 (4) Å	*T* = 294 K
β = 107.392 (4)°	Block, colourless
*V* = 1926.5 (11) Å^3^	0.11 × 0.07 × 0.05 mm
*Z* = 2	

### Data collection

**Table:**

Bruker SMART CCD area-detector diffractometer	4417 independent reflections
Radiation source: fine-focus sealed tube	3343 reflections with *I* > 2σ(*I*)
graphite	*R*_int_ = 0.044
φ and ω scans	θ_max_ = 27.5°, θ_min_ = 2.3°
Absorption correction: multi-scan (*SADABS*; Bruker, 1997)	*h* = −15→15
*T*_min_ = 0.445, *T*_max_ = 0.668	*k* = −18→18
16820 measured reflections	*l* = −15→15

### Refinement

**Table:**

Refinement on *F*^2^	Primary atom site location: structure-invariant direct methods
Least-squares matrix: full	Secondary atom site location: difference Fourier map
*R*[*F*^2^ > 2σ(*F*^2^)] = 0.029	Hydrogen site location: inferred from neighbouring sites
*wR*(*F*^2^) = 0.057	H-atom parameters constrained
*S* = 1.03	*w* = 1/[σ^2^(*F*_o_^2^) + (0.0213*P*)^2^] where *P* = (*F*_o_^2^ + 2*F*_c_^2^)/3
4417 reflections	(Δ/σ)_max_ = 0.001
263 parameters	Δρ_max_ = 0.73 e Å^−^^3^
18 restraints	Δρ_min_ = −0.85 e Å^−^^3^

### Special details

**Table:**

Geometry. All e.s.d.'s (except the e.s.d. in the dihedral angle between two l.s. planes)are estimated using the full covariance matrix. The cell e.s.d.'s are takeninto account individually in the estimation of e.s.d.'s in distances, anglesand torsion angles; correlations between e.s.d.'s in cell parameters are onlyused when they are defined by crystal symmetry. An approximate (isotropic)treatment of cell e.s.d.'s is used for estimating e.s.d.'s involving l.s. planes.
Refinement. Refinement of *F*^2^ against ALL reflections. The weighted *R*-factor *wR* and goodness of fit *S* are based on *F*^2^, conventional *R*-factors *R* are based on *F*, with *F* set to zero for negative *F*^2^. The threshold expression of *F*^2^ > σ(*F*^2^) is used only for calculating *R*-factors(gt) *etc*. and is not relevant to the choice of reflections for refinement. *R*-factors based on *F*^2^ are statistically about twice as large as those based on *F*, and *R*- factors based on ALL data will be even larger.

### Fractional atomic coordinates and isotropic or equivalent isotropic displacement parameters (Å^2^)

**Table:**

	*x*	*y*	*z*	*U*_iso_*/*U*_eq_	
Pb1	0.173727 (14)	0.043850 (12)	0.498903 (14)	0.03372 (6)	
N1	0.2720 (3)	−0.0825 (3)	0.3883 (3)	0.0359 (9)	
N2	0.3507 (3)	0.1041 (3)	0.4296 (3)	0.0311 (8)	
O1	−0.0112 (3)	−0.0419 (2)	0.3725 (3)	0.0457 (8)	
O2	0.0805 (3)	0.0648 (2)	0.2958 (3)	0.0423 (8)	
O3	0.0861 (3)	0.2009 (2)	0.4678 (3)	0.0518 (7)	
O4	0.2321 (3)	0.2249 (2)	0.6272 (3)	0.0544 (8)	
O5	0.3305 (3)	0.3221 (3)	0.8466 (3)	0.0727 (12)	
H1W	0.3119	0.2922	0.7852	0.109*	
H2W	0.4037	0.3247	0.8745	0.109*	
C1	−0.0917 (4)	−0.0099 (3)	0.1737 (4)	0.0316 (10)	
C2	−0.0902 (4)	0.0467 (4)	0.0825 (4)	0.0496 (13)	
H2	−0.0340	0.0953	0.0923	0.059*	
C3	−0.1716 (5)	0.0322 (4)	−0.0240 (4)	0.0610 (16)	
H3	−0.1702	0.0710	−0.0852	0.073*	
C4	−0.2551 (5)	−0.0405 (4)	−0.0383 (5)	0.0591 (15)	
H4	−0.3101	−0.0508	−0.1094	0.071*	
C5	−0.2564 (4)	−0.0974 (4)	0.0529 (4)	0.0517 (14)	
H5	−0.3130	−0.1457	0.0433	0.062*	
C6	−0.1751 (4)	−0.0836 (3)	0.1580 (4)	0.0408 (12)	
H6	−0.1757	−0.1233	0.2187	0.049*	
C7	−0.0022 (4)	0.0053 (3)	0.2875 (4)	0.0358 (11)	
C8	0.2340 (5)	−0.1718 (4)	0.3670 (4)	0.0478 (13)	
H8	0.1693	−0.1909	0.3903	0.057*	
C9	0.2844 (5)	−0.2388 (4)	0.3123 (4)	0.0555 (15)	
H9	0.2538	−0.3008	0.2992	0.067*	
C10	0.3789 (5)	−0.2130 (4)	0.2779 (4)	0.0510 (14)	
H10	0.4144	−0.2572	0.2415	0.061*	
C11	0.4235 (4)	−0.1176 (4)	0.2979 (4)	0.0408 (12)	
C12	0.5245 (5)	−0.0854 (4)	0.2670 (4)	0.0493 (14)	
H12	0.5628	−0.1274	0.2307	0.059*	
C13	0.5649 (4)	0.0053 (4)	0.2898 (4)	0.0467 (13)	
H13	0.6318	0.0242	0.2703	0.056*	
C14	0.5072 (4)	0.0736 (3)	0.3438 (4)	0.0342 (11)	
C15	0.5469 (4)	0.1678 (4)	0.3670 (4)	0.0426 (12)	
H15	0.6125	0.1896	0.3471	0.051*	
C16	0.4878 (4)	0.2280 (4)	0.4197 (4)	0.0429 (12)	
H16	0.5116	0.2917	0.4345	0.051*	
C17	0.3918 (4)	0.1927 (3)	0.4507 (4)	0.0367 (11)	
H17	0.3542	0.2338	0.4885	0.044*	
C18	0.4080 (4)	0.0436 (3)	0.3760 (4)	0.0306 (10)	
C19	0.3653 (4)	−0.0553 (3)	0.3537 (4)	0.0329 (10)	
C20	0.1402 (5)	0.2519 (4)	0.5538 (4)	0.0513 (7)	
C21	0.0877 (5)	0.3482 (4)	0.5642 (4)	0.0572 (11)	
H21A	0.0272	0.3411	0.6012	0.086*	
H21B	0.0535	0.3751	0.4896	0.086*	
H21C	0.1487	0.3902	0.6084	0.086*	

### Atomic displacement parameters (Å^2^)

**Table:**

	*U*^11^	*U*^22^	*U*^33^	*U*^12^	*U*^13^	*U*^23^
Pb1	0.02875 (9)	0.04061 (11)	0.03074 (10)	−0.00348 (9)	0.00726 (7)	−0.00015 (9)
N1	0.036 (2)	0.031 (2)	0.038 (2)	−0.0030 (17)	0.0064 (18)	−0.0009 (17)
N2	0.030 (2)	0.033 (2)	0.030 (2)	−0.0017 (17)	0.0089 (16)	0.0002 (16)
O1	0.0395 (19)	0.065 (2)	0.0321 (18)	−0.0089 (17)	0.0104 (15)	0.0032 (17)
O2	0.043 (2)	0.039 (2)	0.0392 (19)	−0.0107 (15)	0.0051 (16)	0.0036 (15)
O3	0.0595 (17)	0.0502 (16)	0.0416 (15)	0.0045 (13)	0.0091 (13)	−0.0036 (13)
O4	0.0521 (18)	0.0559 (18)	0.0500 (17)	0.0017 (15)	0.0074 (14)	0.0008 (15)
O5	0.052 (2)	0.087 (3)	0.085 (3)	−0.006 (2)	0.029 (2)	−0.008 (2)
C1	0.028 (2)	0.035 (2)	0.030 (2)	0.004 (2)	0.007 (2)	−0.001 (2)
C2	0.046 (3)	0.056 (3)	0.040 (3)	−0.010 (3)	0.004 (2)	0.007 (3)
C3	0.067 (4)	0.073 (4)	0.037 (3)	−0.004 (3)	0.007 (3)	0.010 (3)
C4	0.058 (4)	0.062 (4)	0.041 (3)	0.003 (3)	−0.010 (3)	−0.006 (3)
C5	0.041 (3)	0.047 (3)	0.055 (3)	−0.005 (2)	−0.004 (3)	−0.007 (3)
C6	0.039 (3)	0.038 (3)	0.043 (3)	0.000 (2)	0.009 (2)	0.003 (2)
C7	0.036 (3)	0.038 (3)	0.034 (3)	0.004 (2)	0.011 (2)	−0.004 (2)
C8	0.050 (3)	0.043 (3)	0.045 (3)	−0.011 (3)	0.007 (3)	0.000 (2)
C9	0.075 (4)	0.033 (3)	0.048 (3)	−0.002 (3)	0.002 (3)	−0.007 (3)
C10	0.071 (4)	0.045 (3)	0.031 (3)	0.013 (3)	0.007 (3)	−0.008 (2)
C11	0.047 (3)	0.046 (3)	0.025 (2)	0.015 (2)	0.005 (2)	0.002 (2)
C12	0.051 (3)	0.059 (4)	0.040 (3)	0.025 (3)	0.017 (3)	0.005 (3)
C13	0.039 (3)	0.068 (4)	0.038 (3)	0.018 (3)	0.019 (2)	0.017 (3)
C14	0.024 (2)	0.050 (3)	0.027 (2)	0.006 (2)	0.0062 (19)	0.013 (2)
C15	0.029 (3)	0.057 (3)	0.039 (3)	−0.007 (2)	0.007 (2)	0.013 (2)
C16	0.038 (3)	0.043 (3)	0.047 (3)	−0.012 (2)	0.011 (2)	0.000 (2)
C17	0.034 (3)	0.039 (3)	0.036 (3)	−0.004 (2)	0.009 (2)	−0.002 (2)
C18	0.027 (2)	0.034 (2)	0.027 (2)	0.003 (2)	0.0023 (18)	0.002 (2)
C19	0.035 (2)	0.035 (3)	0.023 (2)	0.007 (2)	−0.0002 (19)	0.005 (2)
C20	0.0571 (16)	0.0509 (16)	0.0431 (15)	0.0041 (13)	0.0105 (13)	−0.0033 (12)
C21	0.062 (2)	0.055 (2)	0.049 (2)	0.0080 (19)	0.0087 (18)	−0.0094 (18)

### Geometric parameters (Å, °)

**Table:**

Pb1—O3	2.399 (3)	C5—H5	0.9300
Pb1—O2	2.426 (3)	C6—H6	0.9300
Pb1—O1	2.565 (3)	C8—C9	1.384 (7)
Pb1—O1^i^	2.828 (3)	C8—H8	0.9300
Pb1—N2	2.619 (4)	C9—C10	1.355 (7)
Pb1—N1	2.688 (4)	C9—H9	0.9300
Pb1—C7	2.851 (5)	C10—C11	1.421 (7)
N1—C8	1.320 (6)	C10—H10	0.9300
N1—C19	1.349 (6)	C11—C19	1.405 (6)
N2—C17	1.321 (5)	C11—C12	1.428 (7)
N2—C18	1.366 (5)	C12—C13	1.348 (7)
O1—C7	1.264 (5)	C12—H12	0.9300
O2—C7	1.261 (5)	C13—C14	1.442 (7)
O3—C20	1.273 (6)	C13—H13	0.9300
O4—C20	1.244 (6)	C14—C15	1.393 (6)
O5—H1W	0.8317	C14—C18	1.407 (6)
O5—H2W	0.8295	C15—C16	1.370 (6)
C1—C2	1.375 (6)	C15—H15	0.9300
C1—C6	1.394 (6)	C16—C17	1.390 (6)
C1—C7	1.495 (6)	C16—H16	0.9300
C2—C3	1.387 (7)	C17—H17	0.9300
C2—H2	0.9300	C18—C19	1.463 (6)
C3—C4	1.387 (7)	C20—C21	1.498 (7)
C3—H3	0.9300	C21—H21A	0.9600
C4—C5	1.377 (7)	C21—H21B	0.9600
C4—H4	0.9300	C21—H21C	0.9600
C5—C6	1.373 (6)		
			
O3—Pb1—O2	71.68 (11)	O1—C7—Pb1	64.1 (2)
O3—Pb1—O1	94.51 (11)	C1—C7—Pb1	176.6 (4)
O2—Pb1—O1	52.41 (10)	N1—C8—C9	124.1 (5)
O3—Pb1—N2	90.27 (12)	N1—C8—H8	118.0
O2—Pb1—N2	77.74 (11)	C9—C8—H8	118.0
O1—Pb1—N2	124.72 (10)	C10—C9—C8	119.2 (5)
O3—Pb1—N1	138.24 (11)	C10—C9—H9	120.4
O2—Pb1—N1	71.93 (11)	C8—C9—H9	120.4
O1—Pb1—N1	78.91 (11)	C9—C10—C11	119.3 (5)
N2—Pb1—N1	62.48 (11)	C9—C10—H10	120.3
O3—Pb1—C7	82.19 (12)	C11—C10—H10	120.3
O2—Pb1—C7	26.08 (12)	C19—C11—C10	116.8 (5)
O1—Pb1—C7	26.33 (11)	C19—C11—C12	120.4 (5)
N2—Pb1—C7	101.47 (13)	C10—C11—C12	122.8 (5)
N1—Pb1—C7	73.94 (12)	C13—C12—C11	120.7 (5)
C8—N1—C19	117.6 (4)	C13—C12—H12	119.7
C8—N1—Pb1	122.9 (3)	C11—C12—H12	119.7
C19—N1—Pb1	119.5 (3)	C12—C13—C14	121.8 (5)
C17—N2—C18	117.6 (4)	C12—C13—H13	119.1
C17—N2—Pb1	121.1 (3)	C14—C13—H13	119.1
C18—N2—Pb1	121.2 (3)	C15—C14—C18	118.6 (4)
C7—O1—Pb1	89.6 (3)	C15—C14—C13	122.6 (5)
C7—O2—Pb1	96.1 (3)	C18—C14—C13	118.8 (5)
C20—O3—Pb1	106.8 (3)	C16—C15—C14	119.0 (4)
H1W—O5—H2W	110.9	C16—C15—H15	120.5
C2—C1—C6	119.3 (4)	C14—C15—H15	120.5
C2—C1—C7	120.2 (4)	C15—C16—C17	119.1 (5)
C6—C1—C7	120.5 (4)	C15—C16—H16	120.5
C1—C2—C3	120.8 (5)	C17—C16—H16	120.5
C1—C2—H2	119.6	N2—C17—C16	124.0 (4)
C3—C2—H2	119.6	N2—C17—H17	118.0
C4—C3—C2	119.4 (5)	C16—C17—H17	118.0
C4—C3—H3	120.3	N2—C18—C14	121.8 (4)
C2—C3—H3	120.3	N2—C18—C19	118.5 (4)
C5—C4—C3	119.7 (5)	C14—C18—C19	119.7 (4)
C5—C4—H4	120.1	N1—C19—C11	123.1 (4)
C3—C4—H4	120.1	N1—C19—C18	118.2 (4)
C6—C5—C4	120.8 (5)	C11—C19—C18	118.7 (4)
C6—C5—H5	119.6	O4—C20—O3	123.1 (5)
C4—C5—H5	119.6	O4—C20—C21	120.1 (5)
C5—C6—C1	119.9 (5)	O3—C20—C21	116.8 (5)
C5—C6—H6	120.0	C20—C21—H21A	109.5
C1—C6—H6	120.0	C20—C21—H21B	109.5
O2—C7—O1	121.9 (4)	H21A—C21—H21B	109.5
O2—C7—C1	118.9 (4)	C20—C21—H21C	109.5
O1—C7—C1	119.2 (4)	H21A—C21—H21C	109.5
O2—C7—Pb1	57.8 (2)	H21B—C21—H21C	109.5
			
O3—Pb1—N1—C8	−124.4 (3)	C6—C1—C7—O1	−8.0 (7)
O2—Pb1—N1—C8	−93.9 (4)	O3—Pb1—C7—O2	63.8 (3)
O1—Pb1—N1—C8	−40.1 (3)	O1—Pb1—C7—O2	−179.1 (5)
N2—Pb1—N1—C8	−179.2 (4)	N2—Pb1—C7—O2	−24.9 (3)
C7—Pb1—N1—C8	−66.7 (4)	N1—Pb1—C7—O2	−81.6 (3)
O3—Pb1—N1—C19	56.5 (4)	O3—Pb1—C7—O1	−117.1 (3)
O2—Pb1—N1—C19	87.0 (3)	O2—Pb1—C7—O1	179.1 (5)
O1—Pb1—N1—C19	140.8 (3)	N2—Pb1—C7—O1	154.2 (3)
N2—Pb1—N1—C19	1.7 (3)	N1—Pb1—C7—O1	97.5 (3)
C7—Pb1—N1—C19	114.2 (3)	C19—N1—C8—C9	0.4 (7)
O3—Pb1—N2—C17	34.5 (3)	Pb1—N1—C8—C9	−178.7 (4)
O2—Pb1—N2—C17	105.7 (3)	N1—C8—C9—C10	0.2 (8)
O1—Pb1—N2—C17	130.2 (3)	C8—C9—C10—C11	−0.5 (7)
N1—Pb1—N2—C17	−178.5 (3)	C9—C10—C11—C19	0.2 (7)
C7—Pb1—N2—C17	116.6 (3)	C9—C10—C11—C12	178.4 (5)
O3—Pb1—N2—C18	−149.1 (3)	C19—C11—C12—C13	−0.6 (7)
O2—Pb1—N2—C18	−77.9 (3)	C10—C11—C12—C13	−178.6 (4)
O1—Pb1—N2—C18	−53.4 (3)	C11—C12—C13—C14	−1.3 (7)
N1—Pb1—N2—C18	−2.1 (3)	C12—C13—C14—C15	−179.3 (4)
C7—Pb1—N2—C18	−67.0 (3)	C12—C13—C14—C18	1.7 (7)
O3—Pb1—O1—C7	62.2 (3)	C18—C14—C15—C16	−0.3 (6)
O2—Pb1—O1—C7	−0.5 (3)	C13—C14—C15—C16	−179.3 (4)
N2—Pb1—O1—C7	−31.3 (3)	C14—C15—C16—C17	1.5 (7)
N1—Pb1—O1—C7	−76.1 (3)	C18—N2—C17—C16	1.3 (6)
O3—Pb1—O2—C7	−110.5 (3)	Pb1—N2—C17—C16	177.8 (3)
O1—Pb1—O2—C7	0.5 (3)	C15—C16—C17—N2	−2.0 (7)
N2—Pb1—O2—C7	155.0 (3)	C17—N2—C18—C14	−0.1 (6)
N1—Pb1—O2—C7	90.3 (3)	Pb1—N2—C18—C14	−176.5 (3)
O2—Pb1—O3—C20	−157.3 (4)	C17—N2—C18—C19	178.9 (4)
O1—Pb1—O3—C20	154.8 (3)	Pb1—N2—C18—C19	2.5 (5)
N2—Pb1—O3—C20	−80.3 (3)	C15—C14—C18—N2	−0.4 (6)
N1—Pb1—O3—C20	−126.8 (3)	C13—C14—C18—N2	178.6 (4)
C7—Pb1—O3—C20	178.1 (4)	C15—C14—C18—C19	−179.4 (4)
C6—C1—C2—C3	1.0 (8)	C13—C14—C18—C19	−0.3 (6)
C7—C1—C2—C3	178.9 (5)	C8—N1—C19—C11	−0.7 (6)
C1—C2—C3—C4	−0.2 (9)	Pb1—N1—C19—C11	178.4 (3)
C2—C3—C4—C5	0.0 (9)	C8—N1—C19—C18	179.6 (4)
C3—C4—C5—C6	−0.6 (9)	Pb1—N1—C19—C18	−1.2 (5)
C4—C5—C6—C1	1.4 (8)	C10—C11—C19—N1	0.4 (6)
C2—C1—C6—C5	−1.6 (7)	C12—C11—C19—N1	−177.7 (4)
C7—C1—C6—C5	−179.5 (4)	C10—C11—C19—C18	−180.0 (4)
Pb1—O2—C7—O1	−1.0 (5)	C12—C11—C19—C18	1.9 (6)
Pb1—O2—C7—C1	179.3 (4)	N2—C18—C19—N1	−0.8 (6)
Pb1—O1—C7—O2	0.9 (5)	C14—C18—C19—N1	178.2 (4)
Pb1—O1—C7—C1	−179.4 (4)	N2—C18—C19—C11	179.6 (4)
C2—C1—C7—O2	−6.3 (7)	C14—C18—C19—C11	−1.4 (6)
C6—C1—C7—O2	171.7 (4)	Pb1—O3—C20—O4	10.0 (7)
C2—C1—C7—O1	174.0 (4)	Pb1—O3—C20—C21	−169.7 (4)

Symmetry codes: (i) −*x*, −*y*, −*z*+1.

### Hydrogen-bond geometry (Å, °)

**Table:**

*D*—H···*A*	*D*—H	H···*A*	*D*···*A*	*D*—H···*A*
O5—H2W···O3^ii^	0.83	2.15	2.958 (5)	166
O5—H1W···O4	0.83	2.11	2.928 (5)	169

Symmetry codes: (ii) *x*+1/2, −*y*+1/2, *z*+1/2.
